# Implication of *FSHB* rs10835638 variant in endometriosis in Brazilian women

**DOI:** 10.31744/einstein_journal/2023AO0483

**Published:** 2023-10-18

**Authors:** Bianca Bianco, Flávia Altheman Loureiro, Camila Martins Trevisan, Denise Maria Christofolini, Antonio Simone Laganà, Caio Parente Barbosa

**Affiliations:** 1 Centro Universitário FMABC Santo André SP Brazil Centro Universitário FMABC, Santo André, SP, Brazil.; 2 Unit of Gynecologic Oncology, ARNAS “Civico-Di Cristina-Benfratelli” Department of Health Promotion, Mother and Child Care, Internal Medicine and Medical Specialties University of Palermo Palermo Italy Unit of Gynecologic Oncology, ARNAS “Civico-Di Cristina-Benfratelli”, Department of Health Promotion, Mother and Child Care, Internal Medicine and Medical Specialties, University of Palermo, Palermo, Italy.

**Keywords:** Endometriosis, Follicle stimulating hormone, beta subunit, Infertility, Polymorphism, single nucleotide

## Abstract

**Objective:**

The follicle-stimulating hormone subunit beta gene rs10835638 variant (c.-211G>T) may have detrimental effects on fertility and protective effects against endometriosis. A case-control analysis was performed, aiming to investigate the possible relationship between this variant and the development and/or progression of endometriosis.

**Methods:**

This study included 326 women with endometriosis and 482 controls without endometriosis, both confirmed by inspection of the pelvic cavity during surgery. Genotyping was performed using a TaqMan real-time polymerase chain reaction assay. Genotype and allele frequencies and genetic models were compared between the groups.

**Results:**

The genotype and allele frequencies of the rs10835638 variant did not differ between women with and those without endometriosis. Subdividing the endometriosis group into fertile and infertile groups did not result in a significant difference in these frequencies. However, the subgroup with minimal/mild endometriosis had a higher frequency of the GT genotype than the Control Group, regardless of fertility. The T allele was significantly more common in women with minimal/mild endometriosis than in the Control Group in the recessive model.

**Conclusion:**

The T allele is associated with the development of minimal/mild endometriosis in Brazilian women.

## In Brief

We performed a case-control study, aiming to investigate the possible relationship between the FSHB rs10835638 variant and the development and/or progression of endometriosis in 326 women with endometriosis and 482 controls. Our results suggest that the T allele is associated with the development of minimal/mild endometriosis in Brazilian women.

## Highlights

First study to investigate the rs10835638 variant in Brazilian women with endometriosis.

The homozygous TT genotype was very rare among Brazilian women.

Genotype frequencies did not differ between endometriosis and Control Groups.

The T allele was more common in minimal/mild endometriosis subgroups than in the Control Group.

## Visual Abstract

**Figure:**
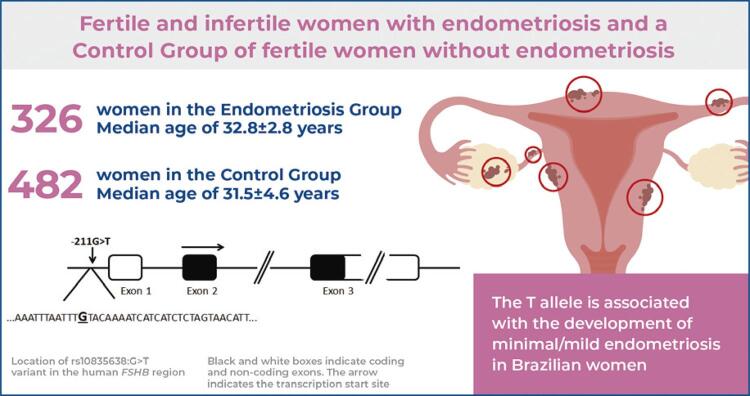

## INTRODUCTION

Endometriosis is an estrogen-dependent, chronic, inflammatory, debilitating disease associated with pelvic pain and infertility. It is characterized by the presence of ectopic endometrial glands and stroma, predominantly but not exclusively in the pelvic compartment. It affects 5%-10% of women of reproductive age^(1-3)^ and can severely compromise their quality of life.^(4)^ Moreover, endometriosis is observed in 50%-80% of women with pelvic pain and in up to 50% of women with infertility.^(5)^

Estrogens fuel ectopic endometrial growth. In addition to the production of estradiol by the ovaries, local production of estradiol in endometriotic lesions is mediated by steroidogenic enzymes, including aromatase.^(6)^ Ectopic endometrial tissue overexpresses estrogen receptor beta (ER-beta, encoded by *ESR2*), resulting in the suppression of estrogen receptor alpha (ER-alpha, encoded by *ESR1*), leading to diminished ER-alpha-mediated induction of the progesterone receptor.^(7)^ Ultimately, ER-beta promotes cell survival and perpetuates inflammation. At the same time as estrogen overproduction and ER overexpression, progesterone resistance occurs in endometriotic tissue, impeding the modulation of genes involved in decidualization, cell cycle regulation, and estrogen response inhibition.^(8)^ Progesterone is a potent antagonist of estrogen-induced proliferation in the endometrium and acts as an anti-inflammatory agent.^(3,9)^

Several theories have been proposed with regards to the possible factors responsible for the development of endometriosis. However, none of the existing theories are able to explain all of the implantation sites and symptoms of the disease. Retrograde menstruation is the pathogenic hypothesis supported by the most robust evidence regarding the mechanism of endometrial peritoneal implants.^(8,10)^

Endometriosis is heritable and is influenced by multiple genetic and environmental factors. Genetic factors contribute to about half of the variation in the endometriosis risk, which has an estimated hereditability of 51%,^(2,11)^ although no distinct inheritance pattern has been established for the disease. Several studies have highlighted the association between single nucleotide variants (SNVs) and an increased risk of endometriosis in populations of different ethnic origins.^(12-15)^ In a UK Biobank cross-sectional study, the T allele of the *FSHB* c.-211G>T polymorphism (rs10835638) was associated with detrimental effects on fertility and protective effects against endometriosis.^(16)^

The β-subunit of the follicle-stimulating hormone (FSH), encoded by the *FSHB* gene, ensures the binding specificity of the hormone to FSH receptors, enabling the hormone to stimulate the growth of ovarian follicles and production of estrogens.^(17,18)^ The abovementioned SNV in the promoter region of the *FSHB* gene reportedly influences assisted reproductive treatments as well as the concentrations of FSH, luteinizing hormone (LH), and progesterone.^(18)^

## OBJECTIVE

To investigate the possible relationship between the *FSHB* gene variant (c.-211G>T, rs10835638) and the development and/or progression of endometriosis.

## METHODS

This case-control study included 326 fertile and infertile women with endometriosis and a Control Group of 482 fertile women without endometriosis. The study design was approved by the independent Research Ethics Committee of *Centro Universitário FMABC* (CAAE: 13974913.4.0000.0082; # 310.094). Each patient enrolled in this study signed an informed consent form for all procedures and for biological sample and data collection and analysis for research purposes.

### Patients

The endometriosis group comprised 326 women (103 fertile and 223 infertile) who underwent *in vitro* fertilization (IVF) treatment at the Human Reproduction and Genetics Center of *Centro Universitário FMABC*, Santo André, Brazil between 2014 to 2019. Inclusion criteria were: age ≤38 years; diagnosis of endometriosis confirmed by laparoscopy or laparotomy and histology, classified according to the revised American Society for Reproductive Medicine (rASRM) score;^(19)^ the presence of both ovaries without morphological abnormalities; ovulatory cycles lasting 25 to 35 days; and no evidence of endocrine diseases, such as hyperprolactinemia, thyroid dysfunction, or polycystic ovary syndrome. Women with a previous ovarian surgery, those who underwent chemo/radiotherapy, and those with endometrial polyps, hydrosalpinx, or submucosal and/or intramural fibroids were excluded from the study.

The investigation of the cause of infertility included hormonal and biochemical profiling, testing for sexually transmitted diseases, imaging examinations, investigation of genetic and/or immunological abnormalities, hysterosalpingography, hysteroscopy, laparoscopy, and semen analysis of the partner.^(20,21)^

We considered women who had undergone laparoscopy/laparotomy to treat endometriosis and who had failed to achieve pregnancy spontaneously or by IVF treatment after surgery within a maximum period of 12 months, as infertile.

The Control Group comprised 482 fertile women who had undergone permanent contraception by laparoscopy. The absence of endometriosis was confirmed by inspection of the pelvic cavity during the procedure, and none of these women had a personal and/or familial history of endometriosis, autoimmune diseases, or cancer.

### Genotyping

Whole-blood samples were collected in EDTA-containing tubes. DNA was extracted from peripheral lymphocytes by using a standard salting-out method.^(22)^ Genotyping of the NG_008144.1:g.4790G>T (c.-211G>T, rs10835638) variant of the *FSHB* gene was performed using the TaqMan system and real-time PCR. Assays were performed using primers, probes, and Master Mix (Thermo Fisher Scientific, Waltham, MA, USA), with 25ng of DNA per reaction. The PCR conditions were as follows: 50 cycles of denaturation at 95°C for 15 seconds, and annealing/extension at 60°C for 90 seconds.

### Statistical analysis

Statistical analyses were performed using the R programming language (R Core Team, 2018).^(23)^ The allele and genotype frequencies were compared between groups by using the χ^2^ test, as well as the estimation of Hardy-Weinberg equilibrium to compare the genetic models (dominant or recessive models) of the SNV. Odds ratios and 95% confidence intervals (CIs) were calculated in relation to the presence of the reference genotype by using a logistic regression model. Statistical significance was set at p<0.05.

## RESULTS

The mean age of the endometriosis group was 32.8±2.8 years; 39.9% had minimal/mild disease, and 60.1% had moderate/severe endometriosis. Moreover, 68.4% of women in this group were considered infertile. The Control Group had a mean age of 31.5±4.6 years.

Genotype and allele frequencies of the *FSHB* rs10835638 variant in women with endometriosis compared to those in controls are summarized in table 1. The genetic models of the variant are summarized in table 2. Only four women with endometriosis and seven in the Control Group presented with the homozygous variant TT genotype. The genotype frequency of the *FSHB* variant was in Hardy-Weinberg equilibrium in all studied groups.

**Table 1:** Genotype and allelic frequencies of the variant follicle stimulating hormone subunit beta rs10835638: G>T in women with endometriosis and controls

Population	n	Genotypes					Alleles			OR (95%CI)	HWE
	
		GG	GT	p value	TT	p value	G	T	p value		
Endometriosis											
Overall	326	259 (79.5)	63 (19.3)	0.060	4 (1.2)	0.862	581 (89.1)	71 (10.9)	0.125	1.30 (0.93-1.81)	0.998
Minimal/Mild	130	96 (73.9)	31 (23.8)	0.007	3 (2.3)	0.388	223 (85.8)	37 (14.2)	0.006	1.76 (1.16-2.67)	0.995
Moderate/Severe	196	163 (83.1)	32 (16.4)	0.536	1 (0.5)	0.314	358 (91.3)	34 (8.7)	0.974	1.01 (0.66-1.53)	0.980
Fertile											
Overall	103	81 (78.6)	20 (19.4)	0.182	2 (2)	0.656	182 (88.3)	24 (11.7)	0.169	1.40 (0.86-2.27)	0.847
Minimal/Mild	49	35 (71.5)	12 (24.5)	0.046	2 (4.0)	0.112	82 (83.7)	16 (16.3)	0.012	2.07 (1.16-3.70)	0.913
Moderate/Severe	54	46 (85.2)	8 (14.8)	0.956	0 (0.0)	0.373	100 (92.6)	8 (7.4)	0.670	0.85 (0.40-1.81)	0.951
Infertile											
Overall	223	178 (79.8)	43 (19.3)	0.099	2 (0.9)	0.592	399 (89.5)	47 (10.5)	0.244	1.25 (0.86-1.82)	0.945
Minimal/Mild	81	61 (75.3)	19 (23.5)	0.036	1 (1.2)	0.964	141 (87.0)	21 (13.0)	0.076	1.58 (0.95-2.63)	0.989
Moderate/Severe	142	117 (82.4)	24 (16.9)	0.467	1 (0.7)	0.505	258 (90.8)	26 (9.2)	0.774	1.07 (0.67-1.70)	0.998
Controls											
	482	406 (84.2)	69 (14.3)	--	7 (1.5)	--	881 (91.4)	83 (8.6)	--	--	0.980

OR: odds ratio; 95%CI: condidence interval; HWE: hardy-weinberg equilibrium.

**Table 2:** Genetic models of the variant rs10835638: G>T of the follicle stimulating hormone subunit beta gene in women with ndometriosis and controls

Population	n	Dominant model				Recessive model			
	
		GG+GT	TT	p value	OR (95%CI)	GG	GT+TT	p value	OR (95%CI)
Endometriosis	326	322	4	0.785	0.84 (0.24-2.90)	259	67	0.080	1.38 (0.96-1.99)
Minimal/Mild	130	127	3	0.494	1.60 (0.41-6.29)	96	34	0.006	1.89 (1.19-30.00)
Moderate/Severe	196	195	1	0.303	0.35 (0.04-2.85)	163	33	0.073	1.08 (0.69-1.69)
Fertile	103	101	2	0.714	1.34 (0.28-6.56)	81	22	0.167	1.45 (0.85-2.47)
Infertile	223	221	2	0.541	0.61 (0.13-2.98)	178	45	0.148	1.35 (0.90-2.03)
Controls	482	475	7		Reference	406	76		Reference

OR: odds ratio; 95%CI: condidence interval.

The genotype and allele frequencies of the *FSHB* rs10835638 variant did not differ between women with and those without endometriosis. Upon subgroup analysis of fertile and infertile women with endometriosis, these frequencies also did not differ. However, the subgroup with minimal/mild endometriosis had a higher frequency of the GT genotype than the Control Group (23.8% *versus* 14.3%, p=0.007). The frequency of the GT genotype was also higher in the subgroup with minimal/mild endometriosis in both fertile (24.5% *versus* 14.3%, p=0.046) and infertile women (23.5% *versus* 14.3%, p=0.036) than that in the Control Group. Regarding the genetic models, the T allele was more common in women with minimal/mild endometriosis than that in the Control Group in the recessive model (p=0.006, 95%CI=1.89 (1.19-3.00).

In the table 3 we presented the frequency of the variant allele T of the *FSHB* rs10835638:G>T SNV in different population. The data was extracted from dbSNP (Database of Single Nucleotide Variants) (https://www.ncbi.nlm.nih.gov/snp/rs10835638) based on the ALFA project (Allele Frequency Aggregator) that presents allele frequency pooled data from dbSNP and summarized results from dbGaP (Database of Genotypes and Phenotypes), which are developed databases and hosted by the of National Center for Biotechnology Information (NCBI) in collaboration with the National Human Genome Research Institute (NHGRI). dbSNP is a free public archive that presents genetic variation within and between different species, while dbGAP contains the results of over 1,200 studies, over 2 million individuals and hundreds of millions of variants, along with thousands of phenotypes and data of molecular assays. The data was also extracted from the ABraOM (Brazilian Online Mutations Archive) repository that contains genomic variants obtained from the exome and complete genome sequencing of SABE (a longitudinal study on Health, Well-Being and Aging) in a census sample of 1,171 elderly people in the city of São Paulo, Brazil. The data presented in table 3 makes clear the diffenrence of allele frequency according to population studied, varying from 0% in African and Latin American to 10.9% in Brazilian people.

**Table 3:** Variant allele frequency in different populations according to the ALFA and ABraOM project

Population	SNV rs10835638:G>T	

	n	VAF
Global	17118	6.5
European	12502	8.1
African	3118	2.1
African american	3010	2.1
Others^†^	108	0.0
Asian	138	1.4
East Asian	114	1.8
South Asian	88	1.0
Other§	24	0.0
Latin American 1^‡^	104	0.0
Latin American 2^#^	456	0.0
ABraOM	1,171	10.8
Present study		
Endometriosis	326	10.9
Control	482	8.6

^†^ Other Africans: individuals of African descent; ^§^ Other Asians: Individuals of Asian descent; ^‡^ Latino-Americans 1: Latin American individuals with Afro-Caribbean ancestry; ^#^ Latin Americans 2: Latin American individuals of primarily European and Native American ancestry.

ALFA projetc: allele frequency aggregator; ABraOM: Brazilian Online Mutations Archive; VAF: variant allele frequency; n: number of genotyped individuals.

## DISCUSSION

To the best of our knowledge, this is the first study in which the relationship between the *FSHB* rs10835638 variant and the development and/or progression of endometriosis was investigated in Brazilian women. The homozygous TT genotype was very rare in this study (a prevalence of 1.4%), detected in only four women in the endometriosis group and seven in the Control Group. The T allele frequency was 10.9% in the endometriosis group and 8.6% in the Control Group, similar to that observed in the European population (14.5%) and higher than that detected in the African population (2.8%).^(24)^ In a study conducted by Polyzos et al.,^(25)^ the prevalence of the TT genotype was 0.5% among a study sample that included women from Europe and Asia; it occurred only in European participants.

In a cross-sectional study by the UK Biobank, that included white British individuals aged 40-69 years, *FSHB* T allele of the rs10835638 variant was tested for associations with mainly female reproductive phenotypes in 119958 individuals (up to 63350 women and 56608 men). Also a GWAS was conducted in 9534 individuals to identify genetic variants associated with length of menstrual cycle. The results showed that the T allele was associated with longer menstrual cycles and increased age at menopause. Moreover, although it showed detrimental effects on fertility, it was protective against endometriosis.^(16)^ On the other hand, in the presente study a case-control analysis was performed to investigate the possible relationship between the *FSHB* variant and the development and/or progression of endometriosis in 808 women who had the the pelvic cavity inspectioned to confirm or exclude endometriosis. The genotypes and allelic frequencies of the *FSHB* variant were not different between women with and without endometriosis, even when the group was subdivided into fertile and infertile. However, considering the stage of the disease, the group with minimal/mild endometriosis had a higher frequency of the GT genotype, regardless of fertility status, and the T allele was more frequent in women with minimal or mild endometriosis than in the Control Group in the recessive model. This is in contrast to the study by Ruth et al.^(16)^which indicated harmful effects of the T variant allele on fertility and protective effects against endometriosis. The results of the present study suggest that the T allele is associated with the development of minimal/mild endometriosis. So, the patients selection was different and also the genetic background of the population studied, as well as the methodological approach. These factors together may be responsible for the discordant findings between the two studies.

*FSHB* encodes the hormone-specific βsubunit of FSH, a key promoter of ovarian follicle growth and estrogen production. The rs10835638 variant is located in the promoter region of the gene, where the transcription factor LHX3 binds, regulating gene expression. *In vitro*, transcriptional activity is reportedly reduced by 50% in cells carrying the T allele.^(26)^ In the study by Schüring et al.,^(18)^ which included 365 women with normal ovulation who underwent IVF treatment owing to male infertility, the T allele was associated with higher FSH and LH concentrations and lower progesterone concentrations. Busch et al.^(27)^ observed that, in healthy peripubertal girls, the presence of the T allele was negatively associated with the number of follicles.

Although the rs10835638 variant potentially decreases the serum FSH concentration, which could alter estrogen production as well as follicular cell growth and dominance, the results of the present study indicate that the genotype and allele frequency of this variant did not differ between fertile and infertile women with endometriosis. However, women with minimal/mild endometriosis had a higher frequency of the T allele than women without endometriosis, regardless of fertility.

The association between endometriosis and infertility is well established, but the mechanisms remain unclear. Severe infiltrative endometriosis is associated with impairment of fertility; however, the mechanism by which such a small lesion, observed during laparoscopy, leads to infertility is unknown.

Recently, Kasapoglu et al.^(28)^ confirmed a faster decline in anti-Müllerian hormone (AMH) concentrations in women with endometriomas who did not undergo surgical or medical therapy than that in healthy women. The median percentage decline in serum AMH concentration after six months of follow-up was 26.4% in the endometrioma group and 7.4% in the Control Group. Moreover, oocyte quality may be affected in women with endometriosis. A study in which women with and those without endometriosis who partook in a donation program were compared revealed that oocytes from donors with endometriosis were associated with significantly lower implantation and pregnancy rates compared with donors with tubal infertility, suggesting an alteration in oocyte quality in women with endometriosis, decreasing implantability.^(29)^ In contrast, Hong et al.^(30)^ compared IVF results between women with a decreased ovarian reserve after surgery for endometrioma and women with a decreased ovarian reserve who did not undergo ovarian surgery; they discovered no difference in FSH or AMH concentrations or antral follicle count in response to controlled ovarian stimulation, nor in assisted reproduction outcomes. Bianco et al.^(31)^evaluated the rs10835638 variant and hormonal profile and reproduction outcomes of 213 women with endometriosis; they discovered that women carrying the T allele had significantly higher serum LH concentrations, but that their assisted reproduction outcomes did not differ from those without it.

As the pathogenesis of endometriosis is highly complex, involving both genetic background and environmental conditions, conflicting results have complicated the interpretation of the relevant data. An advantage of our study was the selection of a homogeneous patient cohort and a Control Group with laparoscopic examination of all patients. With regard to the study limitations, only one *FSHB* variant was was investigated, fertility data were considered, but not hormonal profiles. Moreover, this variant may have a small effect, and its frequency may vary according to the study population.

## CONCLUSION

Our results suggest that the *FSHB* rs10835638 variant is associated with the development of minimal/mild endometriosis in Brazilian women. When approaching a characteristic as complex as endometriosis, the characterization of genotypes that can provide data on predisposition or prognosis are relevant and desirable, even considering the methodological difficulties involved.
